# Supplementary material for: Sondheimer oscillations as a probe of non-ohmic flow in WP2 crystals
Source: Nat Commun. 2021 Aug 10;12:4799. doi: 10.1038/s41467-021-25037-0 (PMC8355329; doi:10.1038/s41467-021-25037-0)
Supplement: Supplementary file 1 — Supplementary Information [file 41467_2021_25037_MOESM1_ESM.pdf]

Sondheimer oscillations as a probe of non-ohmic flow in WP_2 crystals

Maarten R. van Delft[1,*], Yaxian Wang[2], Carsten Putzke[1], Jacopo Oswald[3], Georgios Varnavides[2], Christina A. C. Garcia[2], Chunyu Guo[1], Heinz Schmid[3], Vicky Süss[4], Horst Borrmann[4], Jonas Diaz[1], Yan Sun[4], Claudia Felser[4], Bernd Gotsmann[3], Prineha Narang[2,*], and Philip J.W. Moll[1,*]

[1]Laboratory of Quantum Materials (QMAT), Institute of Materials (IMX), École Polytechnique Fédérale de Lausanne (EPFL), 1015 Lausanne, Switzerland

[2]Harvard John A. Paulson School of Engineering and Applied Sciences, Harvard University, Cambridge, MA 02138, USA.

[3]IBM Research - Zurich, 8803 Rüschlikon, Switzerland

[4]Max Planck Institute for Chemical Physics of Solids, Nöthnitzer Strasse 40, 01187 Dresden, Germany

[*]e-mail: maarten.vandelft@epfl.ch; prineha@seas.harvard.edu; philip.moll@epfl.ch

Supplementary information

Supplementary Note 1: Other Sondheimer orbits in WP_2

As seen in Fig. 2, WP_2 has a Fermi surface (FS) with two spin-split electron and hole pockets. In the main text, we focus our attention on the larger of the dogbone-shaped electron pockets as the most likely origin of the observed Sondheimer oscillations (SO). In principle, however, each of the four pockets may contribute to SO and therefore should be investigated.

In Fig. 3, we show the results of our investigation of each of the three FS pockets not discussed in the main text. In Fig. 3a, b and e, we focus on the smaller dogbone pocket. Similarly to the larger pocket, this pocket exhibits an extended region of near-constant $\frac{dA}{dk}$ which may lead to SO. The average $\frac{dA}{dk}$ in this region is somewhat smaller, leading to a calculated thickness-dependence which makes a less accurate prediction of the experimental data. Nevertheless, we cannot exclude that this pocket also contributes to the observed SO. We do not feed back any experimental data into the calculation. Given the similarity in slope, we could not differentiate between these orbits and expect that in reality, both of them contribute.

Fig. 3c, d, f and g concern the hole cylinders. Here, there are several (periodically repeated) sections of the FS with extremal values of $\frac{dA}{dk}$. Using these values of $\frac{dA}{dk}$ to calculate the thickness dependences of the associated frequencies, we see that most of these orbits do not fit our experimental data. There is only one theoretically possible orbit on the smaller hole cylinder (Fig. 3f) which predicts frequencies in agreement with our experiment. However, it is clear that it cannot be this orbit which we observe. Firstly, because only a narrow slice of the FS can contribute to this orbit, due to the relatively sharp peak in $\frac{dA}{dk}$. The amplitude of any oscillation arising from this FS slice should therefore be approximately 40 times smaller than those arising from the dogbone pockets. Secondly, there is no sign in our data of any of the extremal orbits on the larger hole cylinder (Fig. 3g). If the extremal orbit on the smaller cylinder was observed, then the ones on the larger cylinder should be too. Based on this, it is clear

that the hole cylinders play no role in the observed Sondheimer oscillations.

Supplementary Note 2: Quantum oscillations

Here we discuss the quantum oscillations (SdH) observed in our samples (see Fig. 4.) and extract a quantum lifetime τ_q as well as effective masses for several orbits.

These QO can be observed as weak $1/B$-periodic oscillations of the longitudinal resistivity above 14 T, shown in Fig. 4a. In order to separate the oscillatory part of the signal from the large background, we take the second derivative of the data and plot it against $1/B$. From the resulting signal, we then extract the Fast Fourier Transform shown in Fig. 4b. The effective masses listed in this figure were extracted through a fitting of the peak amplitudes as a function of temperature, using the Lifshitz-Kosevich (LK) formula.

In Fig. 4c, we show a Dingle plot for a QO orbit with a frequency of 2.6 kT at a temperature of 1.5 K. This plot is made by taking the amplitudes of the peaks in the oscillatory component of the resistivity acquired after subtraction of a smooth background, $\Delta\rho_{xx}$, and dividing by the thermal damping term from the LK formula, R_T. Plotting the logarithm of this against the inverse of the magnetic field yields a linear relation with slope $14.69\frac{m^*}{m_e}T_D$, where $T_D = \frac{\hbar}{2\pi k_B \tau_\mathrm{q}}$. From this, we find $\tau_\mathrm{q} = (3 \pm 1) \times 10^{-13}$ s. This is a typical value for τ_q, which in our measurements ranges from 10^{-13} s to 10^{-12} s for different QO frequencies, temperatures and devices.

We have also studied the QO as a function of the angle between the magnetic field and the device. The results of this study are shown in Fig. 4d and are in good agreement with previously published theoretical calculations as well as data[1].

References

1. Schönemann, R. *et al.* Fermi surface of the Weyl type-II metallic candidate WP_2. *Phys. Rev. B* **96**, 12110(R), (2017).
2. Kokalj, A. XCrySDen-a new program for displaying crystalline structures and electron densities. *J. Mol. Graph. Model.* **17**, 176–179, (1999).

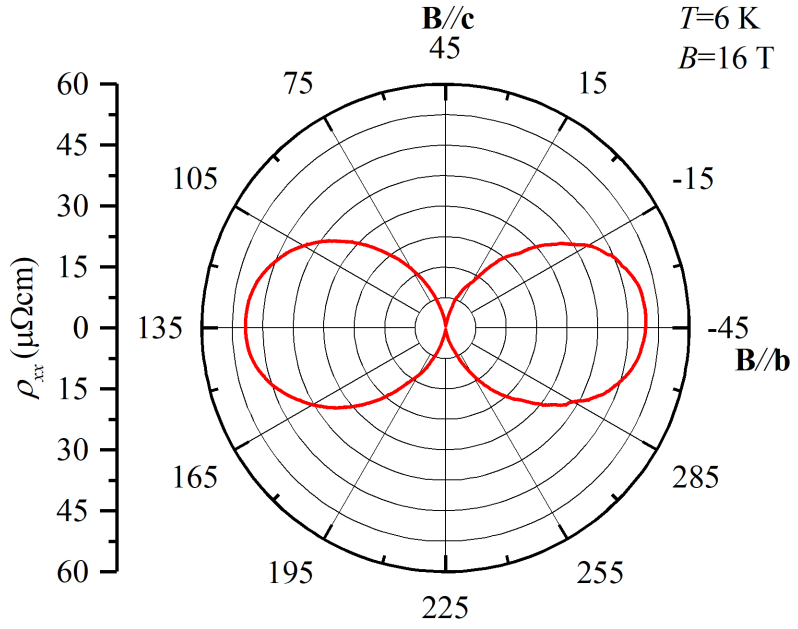

Supplementary Fig. 1. Angle-dependent magnetoresistance of a WP_2 device. Here, 90° is perpendicular to the plane of the sample and high symmetry directions are indicated.

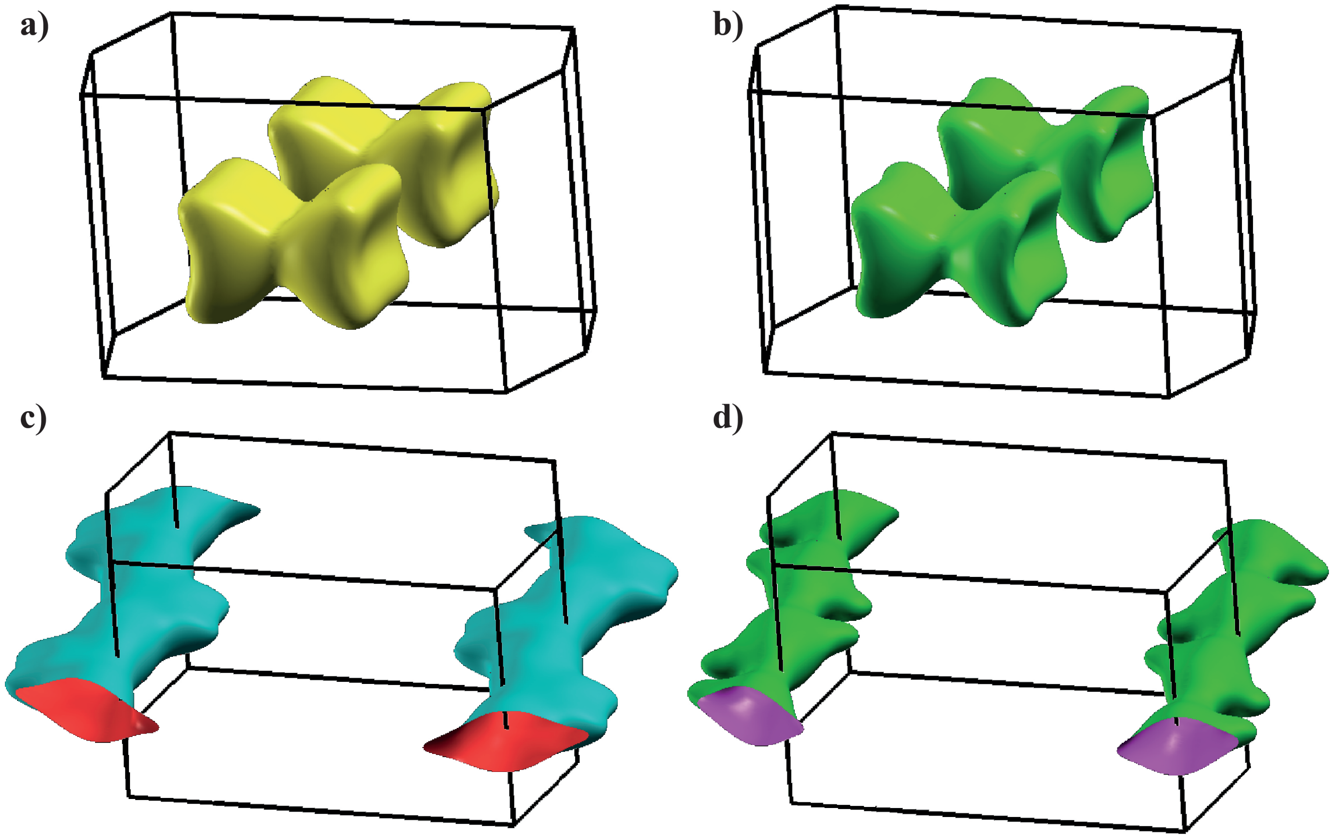

Supplementary Fig. 2. Fermi surface of WP_2. a and **b**, Spin-split electron pockets. **c** and **d**, Spin-split hole pockets. Figure made with XCrySDen[2].

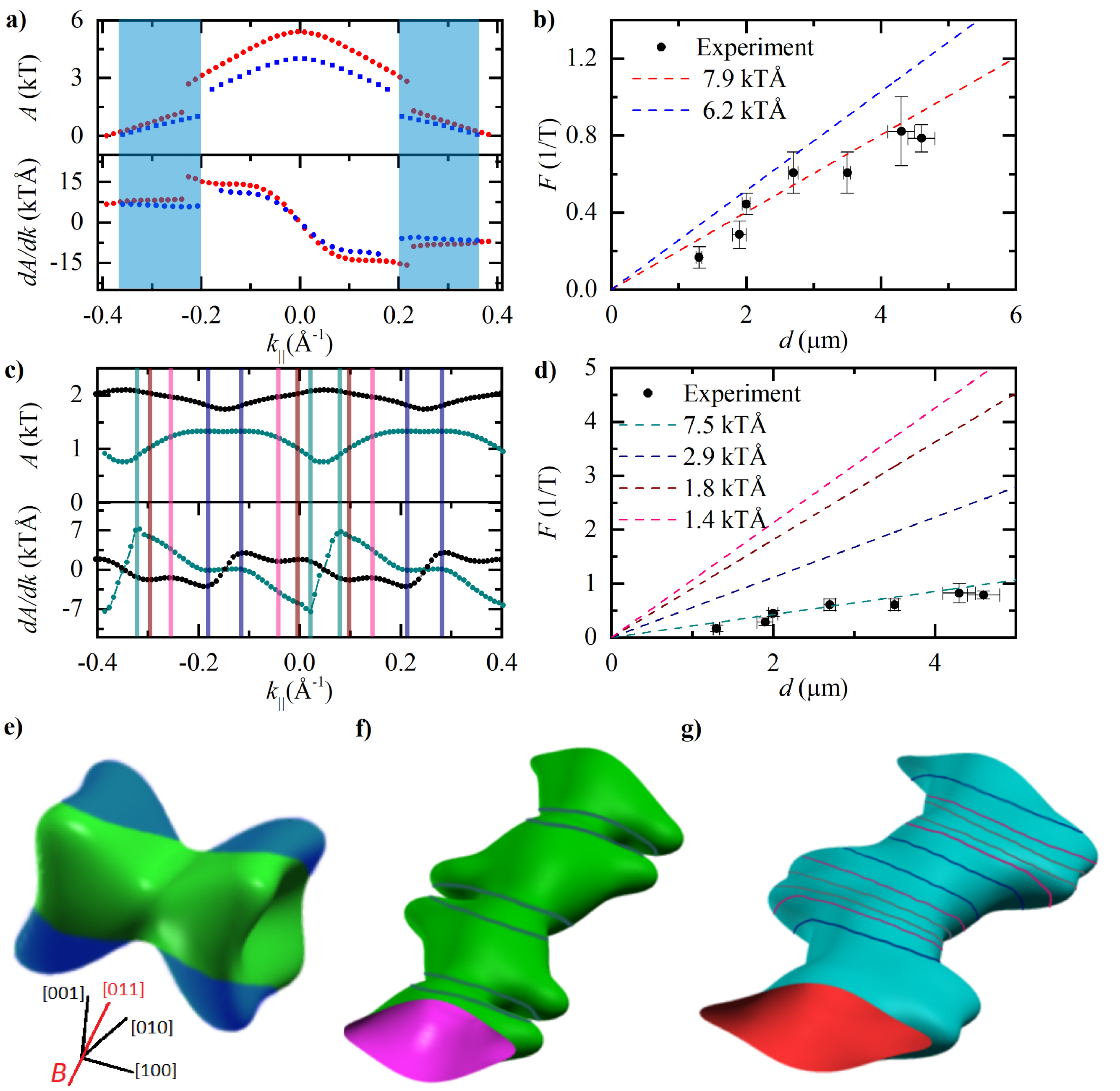

Supplementary Fig. 3. Overview of all possible Sondheimer orbits in WP_2 with a magnetic field along the [011] direction. **a** and **c**, cross-sectional area as a function of $k_{||}$ (top) and its derivative (bottom). In **a**, both dogbone-shaped electron pockets, with the possibly Sondheimer-active region indicated for the smaller pocket (blue); in **c**, the two hole pockets, with locations of possible Sondheimer orbits indicated. **b** and **d**, calculated slopes of the SO frequency against the sample thickness for the orbits indicated in **a** and **c** respectively. All plots include the experimental data (the same as in main Fig. 3) and **b** also includes the calculation for the larger dogbone (red), for comparison. **e**, **f** and **g**, the approximate orbits drawn onto the FS pockets. The directions of the crystallographic axes as well as the magnetic field are indicated in **e**.

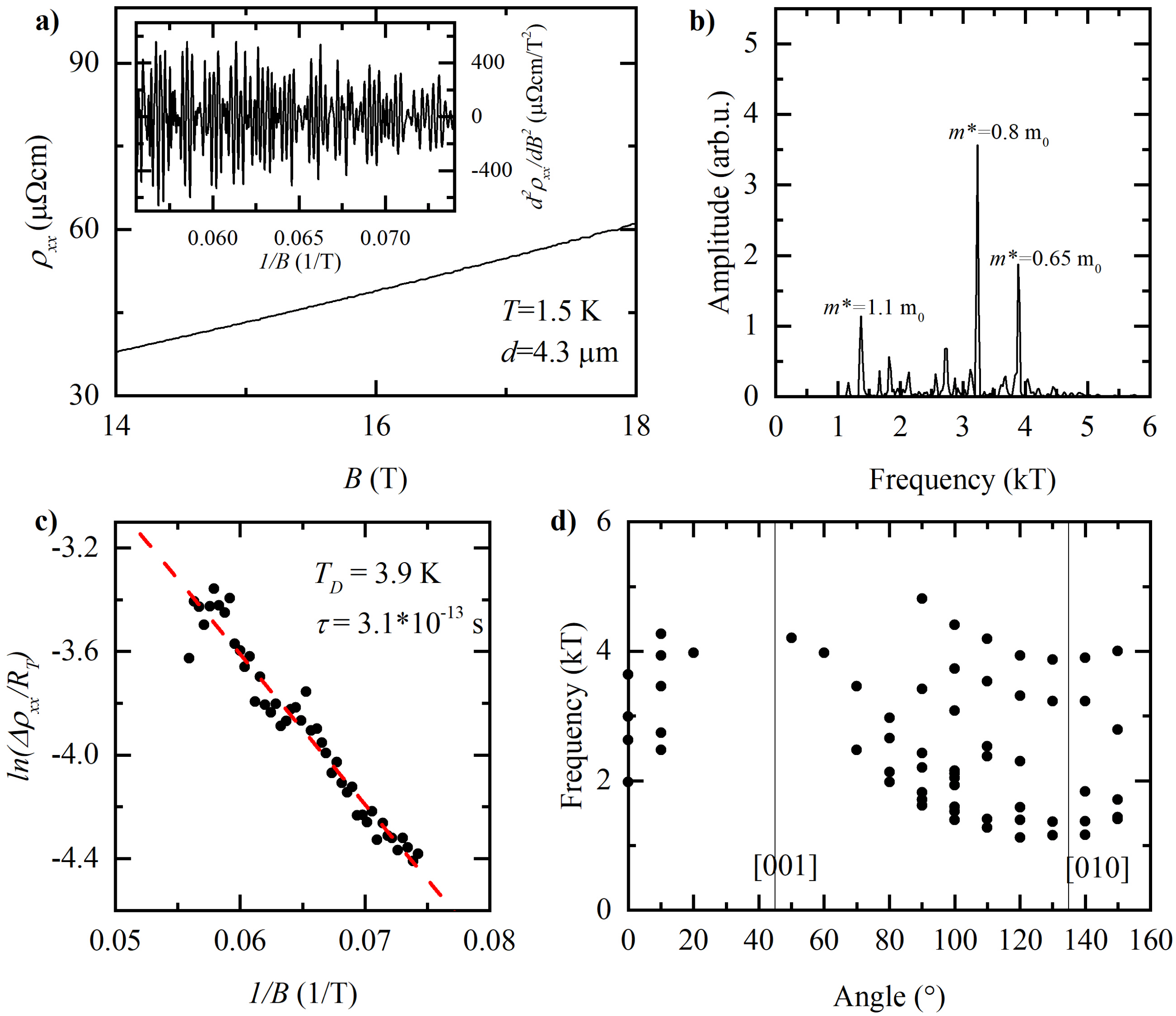

Supplementary Fig. 4. Quantum oscillations in WP_2 microdevices. a, Resistivity of one of the WP_2 microdevices, focused on the region of 14 to 18 T. Inset: Second derivative to B of the data with respect to the magnetic field, plotted against $1/B$ in order to highlight the quantum oscillations. **b**, FFT of the data in **a**, with the effective mass indicated for certain frequencies. **c**, Dingle plot for the QO with frequency of 2.6 kT. **d**, Dependence of the oscillation frequencies between the sample and magnetic field. Here, 90° is perpendicular to the plane of the sample. High symmetry directions are indicated.

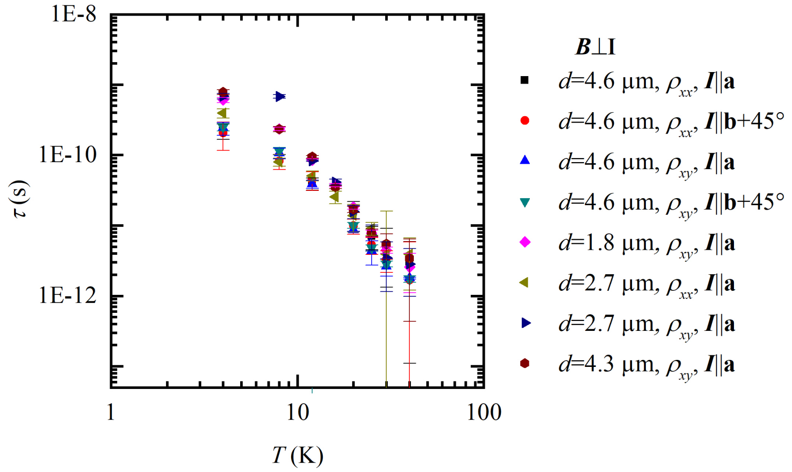

Supplementary Fig. 5. Reproducibility of the extracted lifetimes. Momentum relaxing scattering lifetime extracted from the Sondheimer oscillation amplitude for several different device configurations, showing the reproducibility and robustness of the method. Device thickness, channel (Hall or longitudinal) and current direction (along the crystallographic *a*-axis, or between the *b*- and *c*-axes) do not affect the measured lifetime.
